# Effects of Intraosseous Erythropoietin during Hemorrhagic Shock in Swine

**DOI:** 10.1371/journal.pone.0110908

**Published:** 2014-11-03

**Authors:** Vesna Borovnik-Lesjak, Kasen Whitehouse, Alvin Baetiong, Yang Miao, Brian M. Currie, Sathya Velmurugan, Jeejabai Radhakrishnan, Raúl J. Gazmuri

**Affiliations:** 1 Resuscitation Institute at Rosalind Franklin University of Medicine and Science, North Chicago, Illinois, United States of America; 2 Department of Medicine and Resuscitation Institute at Rosalind Franklin University of Medicine and Science, North Chicago, Illinois, United States of America; 3 Department of Medicine, Department of Physiology and Biophysics, and Resuscitation Institute at Rosalind Franklin University of Medicine and Science, and Critical Care Medicine at the Captain James A. Lovell Federal Health Care Center, North Chicago, Illinois, United States of America; Georgia Regents University, United States of America

## Abstract

**Objective:**

To determine whether erythropoietin given during hemorrhagic shock (*HS*) ameliorates organ injury while improving resuscitation and survival.

**Methods:**

Three series of 24 pigs each were studied. In an initial series, 50% of the blood volume (BV) was removed in 30 minutes and normal saline (threefold the blood removed) started at minute 90 infusing each third in 30, 60, and 150 minutes with shed blood reinfused at minute 330 (*HS-50_BV_*). In a second series, the same *HS-50_BV_* protocol was used but removing an additional 15% of BV from minute 30 to 60 (*HS-65_BV_*). In a final series, blood was removed as in *HS-65_BV_* and intraosseous vasopressin given from minute 30 (0.04 U/kg min^−1^) until start of shed blood reinfusion at minute 150 (*HS-65_BV_+VP*). Normal saline was reduced to half the blood removed and given from minute 90 to 120 in half of the animals. In each series, animals were randomized 1∶1 to receive erythropoietin (1,200 U/kg) or control solution intraosseously after removing 10% of the BV.

**Results:**

In *HS-50_BV_*, O_2_ consumption remained near baseline yielding minimal lactate increases, 88% resuscitability, and 60% survival at 72 hours. In *HS-65_BV_*, O_2_ consumption was reduced and lactate increased yielding 25% resuscitability. In *HS-65_BV_+VP*, vasopressin promoted hemodynamic stability yielding 92% resuscitability and 83% survival at 72 hours. Erythropoietin did not affect resuscitability or subsequent survival in any of the series but increased interleukin-10, attenuated lactate increases, and ameliorated organ injury based on lesser troponin I, AST, and ALT increases and lesser neurological deficits in the *HS-65_BV_+VP* series.

**Conclusions:**

Erythropoietin given during HS in swine failed to alter resuscitability and 72 hour survival regardless of HS severity and concomitant treatment with fluids and vasopressin but attenuated acute organ injury. The studies also showed the efficacy of vasopressin and restrictive fluid resuscitation for hemodynamic stabilization and survival.

## Introduction

Acute hemorrhage resulting from traumatic injury is responsible for a high percentage of death in military personnel engaged in combat operations [1]. A recent report including 4,596 battlefield fatalities from Operation Iraqi Freedom and Operation Enduring Freedom between October 2001 and June 2011 showed that 87.3% of all injury related deaths occurred before arriving to a medical treatment facility [2]. Of these deaths, 24.3% were deemed potentially survivable with acute mortality associated with hemorrhage in 90.9%. The current acute management of hemorrhage focuses on hemostasis, hemodynamic stabilization, and rapid transfer to a medical treatment facility.

Erythropoietin (EPO) − a hormone best known for its effect on red blood cell production − has been shown to protect organs and tissues from ischemia and reperfusion injury including the heart [3]–[7], brain [8], [9], spinal cord [10], [11], kidney [12]–[14], liver [13]–[15], and skin [16], [17]. We have reported beneficial effects of EPO for resuscitation from cardiac arrest in animal models [18]–[20] and in human victims of sudden cardiac arrest [21]. These effects were in part associated with non-genomic activation of mitochondrial protective pathways (e.g., Akt and PKC_ε_) leading to lesser myocardial injury and dysfunction during and after resuscitation [20]. We hypothesized that similar benefits could be elicited in other low-flow states such as hemorrhagic shock (HS) and ameliorate organ injury improving resuscitability and survival. This hypothesis was supported by rat models of HS in which pretreatment with EPO improved survival associated with lesser reductions in mean aortic pressure and lesser increases in lactic acid, tumor necrosis factor (TNF)-α, and interleukin (IL)-6 [14] along with lesser injury to the liver and kidneys [13], [14], and by studies – also in rats – showing that EPO given during HS attenuated intestinal mucosal injury and bacterial translocation [22] along with maintaining intestinal microcirculatory blood flow [23]. Although – to the best of our knowledge – the effects of EPO during HS have not been investigated in large animal models (i.e., swine, sheep, and dog), EPO has been shown to exert tissue protection in swine models of liver [15] and spinal cord [11] ischemia.

We developed a model of HS in swine – an animal higher in the phylogenic scale and thus of greater translational relevance – and investigated the effects of EPO incorporating logistic constraints expected to limit care in far forward combat operations. We used a protocol of controlled bleeding as the initial approach in a multi-year project to first characterize the effects of the proposed interventions without the confounding elements of uncontrolled bleeding and tissue injury (to be incorporated in future series). We conducted three successive series of 24 animals each in which animals were randomized 1∶1 to receive EPO (1,200 U/kg) or control solution. The series had in common (a) removal of blood to a target percentage of the estimated blood volume (simulating bleeding and hemostasis in the field); (b) delivery of EPO through the intraosseous route upon removal of 10% of the animal's blood volume (simulating early drug delivery using a low-skill technique); (c) fluid resuscitation with 0.9% NaCl (normal saline) initiated after a period of untreated HS (simulating delayed access to rescuers); (d) shed blood reinfusion at the end of HS (simulating arrival to a medical post), and (e) contingent on the series, recovery from anesthesia and 72 hour observation. The first series modeled low severity HS; the second series modeled high severity HS; and the third series modeled high severity HS with use of vasopressin to augment resuscitability while examining the role of limited fluid resuscitation.

## Materials and Methods

The studies were approved by the Institutional Animal Care and Use Committee (IACUC) at Rosalind Franklin University of Medicine and Science (approval number 12–23) and by the United States Army Medical Research and Materiel Command Animal Care and Use Review Office (ACURO) and were conducted according to institutional guidelines.

### Animal Housing and Husbandry

Animals were group housed in pens at the Biological Resource Facility (AAALAC accredited facility) at the Rosalind Franklin University of Medicine and Science in which lights are set at the recommended illumination levels of a 12/12-hour cycle controlled via automatic timers. Temperature was maintained at 61–81°F. Resting mats were provided and Aspen Sani-Chip bedding from a certified vendor (Harlan Laboratories, Indiana) was used. Health assessment for general health and well-being, possible injuries, or death was performed daily by animal care technicians and the day before/during/after the experiment by the investigator.

### Animal Preparation

#### Basic Preparation

**Figure 1:**
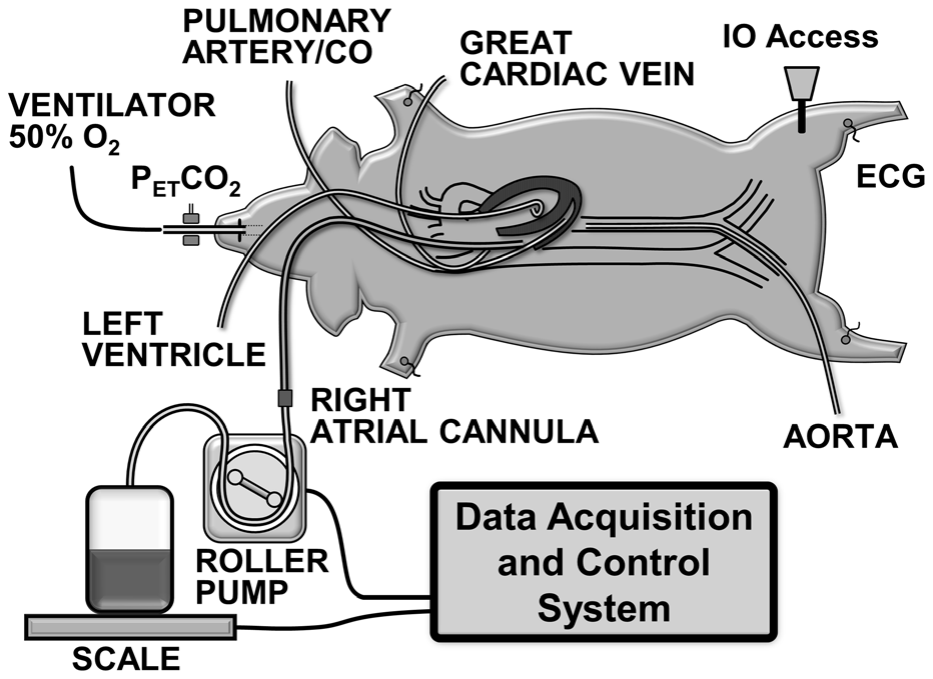
Swine model of hemorrhagic shock. CO, cardiac output; IO, intraosseous; P_ET_CO_2_, end-tidal PCO_2_; ECG, electrocardiogram.

Male domestic pigs (32–48 kg, age ∼11 weeks) were sedated with ketamine hydrochloride (30 mg·kg^−1^ intramuscularly). Anesthesia was induced with propofol (2 mg·kg^−1^ through an ear vein) and the animal intubated with a size 8 tracheal tube initiating positive pressure ventilation with a volume controlled ventilator (840 Ventilator System, Nellcor Puritan Bennett, Boulder, CO) set to deliver a tidal volume of 10 mL·kg^−1^, peak flow of 60 l·min^−1^, and FiO_2_ of 0.5. Respiratory rate was adjusted at baseline to maintain the end-expired PCO_2_ (P_ET_CO) between 35 and 45 mmHg (Capnogard, Novometrix Medical Systems, Wallingford, CT). Anesthesia was continued using isoflurane (1.75% to 2.75%) and a 1∶1 mixture of nitrous oxide and oxygen. The electrocardiogram was recorded through defibrillator adhesive skin pads. All procedures were performed using sterile technique. A 7 F high-fidelity micro-tip catheter transducer (Millar Instruments, Houston, TX) was advanced through the right femoral artery into the descending thoracic aorta for pressure measurement (Figure 1). A 7 F thermodilution balloon-tipped pulmonary artery catheter was advanced through the left cephalic vein or through the left internal jugular vein (when the cephalic vein was used to access the great cardiac vein as described under *Experimental Series*) into the pulmonary artery for measuring core temperature and thermodilution cardiac output along with pressures in the right atrium and pulmonary artery. A 6 F high-fidelity micro-tip pressure transducer pigtail catheter (Millar Instruments, Houston, TX) was advanced through the surgically exposed left carotid artery for measuring left ventricular pressure. A 23 F cannula (Bio-Medicus, Medtronic, Minneapolis, MN) was advanced through the left external jugular vein into the right atrium and used for blood withdrawal into a blood transfer bag. Core temperature was maintained between 37.5°C and 38.5°C with water-circulated blanket (Blanketrol II, Cincinnati SubZero, Cincinnati, OH).

### Hemorrhagic Shock Protocol

The animal's blood volume was estimated at 60 ml/kg-body weight and a predetermined percentage was withdrawn into a heparinized transfer bag (heparin 10 U·ml^−1^ of blood) using a roller pump (model 313S, Watson Marlow, Inc., Wilmington, MA) controlled by a custom-developed software in LabVIEW 6.0. The heparinized transfer bag was placed on an electronic scale (model 2200, Doran Scales, Inc., Batavia, IL) connected to the LabVIEW software to gravimetrically monitor the rate of blood withdrawal (blood density  = 1.06 g/ml) and automatically adjust the pump rate as needed (Figure 1). The withdrawn blood was kept in a water bath at 37.5°C until reinfusion. Resuscitation was subsequently attempted by administration of normal saline followed by reinfusion of the shed blood using a blood transfusion filter (PALL Biomedical, Port Washington, NY). The volume, timing, and use of additional drugs varied as described under *Experimental Series*. In each series, pigs were randomized 1∶1 to receive a 1,200 U/kg bolus of erythropoietin (Epogen [epoetin alpha]; 20,000 U/ml, Amgen) or normal saline vehicle (control) into the left tibia upon 10% removal of the blood volume (6 minutes from the start of blood removal). The investigators were blind to the treatment assignment and the group identification was revealed only after completion of the data analysis in each series.

At the completion of resuscitation in the first and third series, all catheters were removed, vessels ligated, and the skin wounds stapled, all under sterile conditions. The animal was allowed to recover from anesthesia and the endotracheal tube removed after resumption of spontaneous breathing and returned to its pen. The animal was then monitored every 60 minutes until it was able to right itself to sternal recumbency and thereafter every 4 hours for the initial 24 hours and at a minimum interval of 8 hours until completion of the 72 hours. A fentanyl dermal patch was used for analgesia throughout the 72 hour post-resuscitation period. If additional analgesia was needed, 2.2 mg/kg of flunixin meglumine was administered intramuscularly. The neurological status was evaluated at 24, 48, and 72 hours post-resuscitation using a neurological deficit score (0 =  best; 420 =  worst) [24]. The pig was euthanized at 72 hours by intravenous injection of euthanasia solution (pentobarbital sodium and phenytoin sodium; 5 ml, Vedco Inc., St Joseph, MO), or earlier – for humanitarian reason – in the event of moderate to severe of pain and distress unalleviated by analgesic agents, inability to eat or drink unassisted after 24 hours post-surgery, non-weight bearing or paralysis after 24 hours, depression or lethargy after 48 hours, profuse diarrhea, infection not resolved with antimicrobial therapy, lack of righting reflex, or cyanosis with difficulty breathing. The choice of drugs, route of administration, surgical preparation, and method of euthanasia were based on the recommendations by ACLAM board certified DVMs.

After euthanasia, the whole left lung was weighed before and after drying in the oven at 70°C for at least 72 hours for calculations of the wet/dry ratio in the last series.

### Sample Size

The sample size of 12 pigs per group was based on extrapolation from work in similar animal models intended to identify biologically robust differences in survival effects and continuous variables with a power>0.60 at an α level of 0.05.

### Experimental Series

**Figure 2:**
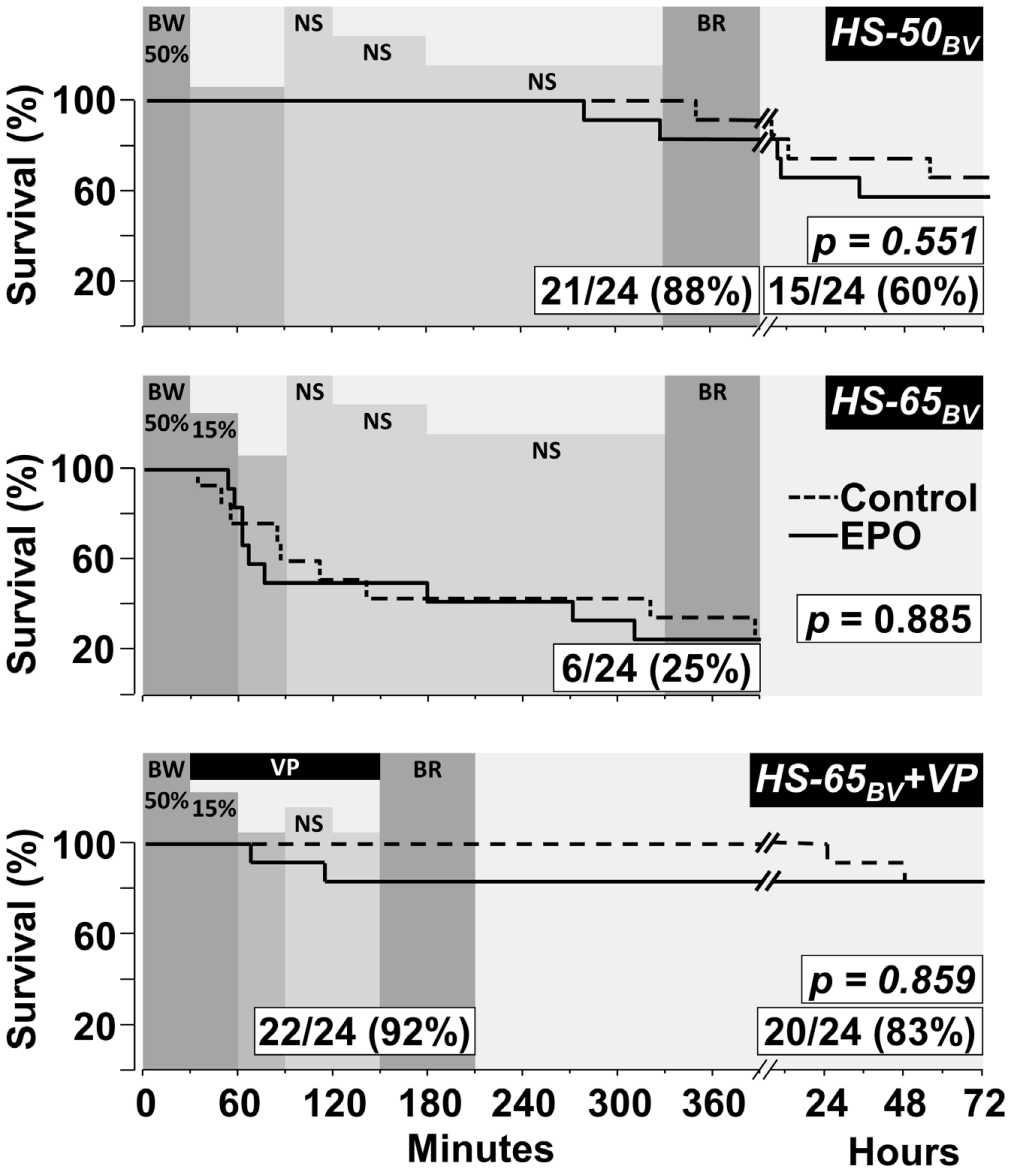
Survival curves comparing pigs treated with EPO and controls. Series *HS-50_BV_* and *HS-65_BV_+VP* include 72 hour survival. The *p*-values for survival differences between groups were calculated using the Gehan-Breslow test and are shown within each graph along with the resuscitation and survival rates for the combined EPO and control groups. The shaded horizontal bars successively represent; the percentage of blood withdrawn (BW), the interval of hemorrhagic shock after blood withdrawal without fluid administration, the administration of normal saline (NS) as described in the Method, and blood reinfusion (BR). Shown in *65_BV_+VP* is the vasopressin infusion (VP).

Experiments were performed between 9 AM to 5 PM in a large animal surgical suite located inside the university Biological Resource Facility. Three consecutive series of 24 experiments each were conducted. The sequence of interventions are described below and depicted in Figure 2. In the first series, 50% of the estimated blood volume was withdrawn in 30 minutes (*HS-50_BV_*). Animals remained untreated for 60 minutes after which normal saline – threefold the blood volume removed – was infused delivering sequentially a third of each in 30, 60, and 150 minutes followed by infusion of the shed blood in 60 minutes. The *HS-50_BV_* protocol triggered a vigorous adaptive response that enabled maintaining oxygen consumption close to baseline resulting in minimal lactate increases and high resuscitability and survival without differences between EPO and control. To test EPO under greater HS severity, a second series was conducted withdrawing an additional 15% of the blood volume in 30 minutes after completing the initial 50% of blood volume removal for a total of 65% of blood volume removed (*HS-65_BV_*). Animals remained untreated for 30 minutes after which the same *HS-50_BV_* protocol for fluid resuscitation and blood reinfusion was applied. In this series, an additional 7 F angiographic catheter was advanced with the aid of fluoroscopy from the left cephalic vein into the great cardiac vein to assess effect on myocardial metabolism [25]. The *HS-65_BV_* protocol was indeed severe, reducing resuscitability to only 25%, but again showing no difference between EPO and control. A third series was then conducted using the same *HS-65_BV_* protocol for blood withdrawal but infusing arginine vasopressin to prevent death by maintaining a higher coronary perfusion (*HS-65_BV_+VP*). Vasopressin (Pitressin, JHP Pharmaceuticals, Rochester, MI) was given intraosseously as a bolus (0.04 U·kg^−1^) upon completion of the initial 50% of blood volume removal followed by a continuous infusion (0.04 U·kg^−1^·min^−1^) using a syringe pump (PHD 2000 Syringe Pump Series, Harvard Apparatus, Holliston, MA) until start of blood reinfusion.

In *HS-65_BV_+VP*, we also assessed the effect of less or no fluid resuscitation [26] under conditions of shorter HS duration. Thus, animals were also randomized 1∶1 to receive either normal saline infusion – half of the blood volume withdrawn in 30 minutes – or no fluid at all. Blood was reinfused starting at 150 minutes (Figure 2). The addition of vasopressin dramatically improved resuscitability, allowing examination of survival and impact on organ function by blood sampling every 24 hours from the superior vena cava after sedation with ketamine hydrochloride (30 mg·kg^−1^ intramuscularly). Pigs were euthanized at 72 hours.

### Experimental Outcomes

The primary outcome was survival at 390 minutes in series *HS-65_BV_* (without recovery from anesthesia) and survival at 72 hours in series *HS-50_BV_* and in series *HS-65_BV_+VP* (with recovery from anesthesia). Secondary outcomes included: (1) hemodynamic and metabolic function, (2) myocardial function, (3) organ injury including the heart, brain, lung, liver, and kidney, (4) plasma cytokines, and (5) blood cell count.

### Measurements

#### Blood analysis

Blood samples were collected from the aorta and pulmonary artery in all three series with the addition of great cardiac vein in *HS-65_BV_*. Blood samples were processed on site for pH, PO_2_, PCO_2_, hemoglobin, and lactate using a cartridge based device (OPTI CCA-TS Blood Gas and Electrolyte Analyzer, OPTI Medical Systems, Roswell, GA) and for common hemoglobin types (oxy-, met-, carboxy-, and reduced-) using a co-oximeter (AVOXimeter 4000, AVOX systems Inc., San Antonio, TX). O_2_ content in the aorta (CaO_2_), pulmonary artery (CvO_2_), and great cardiac vein (CgcvO_2_) was calculated according to the following equation:



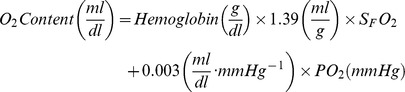
where 1.39 denotes ml of O_2_ bound to 1 g of hemoglobin (Hufner's number), S_F_O_2_ the fraction of oxyhemoglobin relative to the four hemoglobin types, and 0.003 the O_2_ solubility coefficient. Aortic blood samples were also taken and processed for complete blood count and chemistry (blood urea nitrogen [BUN], creatinine, alanine aminotransferase [ALT], aspartate aminotransferase [AST], and troponin I) at the Captain James A. Lovell Federal Health Care Center, North Chicago, IL.

#### Plasma EPO

In series *HS-50_BV_*, the serum level of EPO was measured in three animals that received EPO and in one control using a double-antibody “sandwich” enzyme-linked immunosorbent assay kit (MD Bioproducts, St Paul, MN) targeted to human EPO according to the manufacturer instructions. The EPO level in serum samples (diluted 100 times) was calculated using a standard curve generated with EPO calibrators included in the kit (0, 10.3, 24.8, 48, 156, and 523 mU/ml). The final plasma concentration was determined by applying the dilution factors and a conversion factor whereby one U/ml of EPO [Epogen (epoetin alpha), Amgen] equaled 0.798 U/ml of the calibrator.

#### Hemodynamic Measurements

Thermodilution cardiac output was measured in duplicate after bolus injection of normal saline (5 ml) into the right atrium (HP-Philips M012AT cardiac output module, Amsterdam, The Netherlands). Cardiac output was normalized to body surface area using the Kelley equation (body surface area [m^2^]  = 0.073·body-weight^2/3^ [kg]) [27]. Aortic and left ventricular pressure signals were calibrated with a built-in calibration system (PCU-2000, Millar). Other pressure signals were zeroed to mid-cavity level. All signals were sampled and digitized at 250 Hz using a 16-bit data acquisition board (AT-MIO-16XE-50; National Instruments, Austin, TX) and analyzed using custom developed software (Labview 6.0, National Instruments).

#### Cytokine Measurements

In the *HS-65_BV_+VP* series, plasma levels of IL-6, IL-8, IL-10, and TNF-α were measured by a prototype 4-plex porcine cytokine electrochemiluminescence assay kit (Lot# Z00X2801, Meso Scale Discovery) using a QuickPlex SQ 120 multiplex imager (Meso Scale Discovery). The assay was performed as recommended by the manufacturer. Standards were prepared using IL-6, IL-8, IL-10, and TNF-α calibrators provided in the assay kit after a series of dilutions representing concentrations of 10,000 pg/ml, 2500 pg/ml, 625 pg/ml, 156.3 pg/ml, 39.1 pg/ml, 9.8 pg/ml, 2.4 pg/ml, and 0 pg/ml. Plasma collected at baseline, end of blood withdrawal, 24 hours after resuscitation, and 72 hours after resuscitation previously stored at −80°C was thawed in ice and centrifuged at 2,320 *g* for 10 minutes. Twenty five microliters of the plasma was used for the assay. All standards and samples were run in duplicates. Concentrations were calculated from a 4-parameter logistic equation standard curve using Discovery Workbench software (Meso Scale Discovery). Lower limit of detection (LLOD) of the assay was 2 pg/ml for IL-6, 5 pg/ml for IL-8, 1 pg/ml for IL-10, and 4 pg/ml for TNF-α. The coefficient of variation between the duplicate samples was <5%.

#### Cardiac Function

Indices of cardiac function were derived from left ventricular pressures, reporting the maximal rate of left ventricular pressure increase (dP/dt_max_) and pressure decrease (dP/dt_min_), the stroke volume index (SVI), and the left and right ventricular stroke work (LVSWI and RVSWI), corresponding to SVI times the difference between the systolic and end-diastolic left ventricular pressures (LVSWI) and SVI times the difference between the mean pulmonary and right atrial pressures (RVSWI) expressed in centijoules (cJ) by multiplying by 0.013332 [28].

### Statistical Analysis

SigmaPlot 11.0 (Systat Software, Point Richmond, CA) was used for statistical analysis. For all repetitive variables, two-way repeated measures ANOVA was used to test for the treatment effect between groups and their interaction over time identifying differences at specified time points when present. For clarity, statistical results are presented only at time points shown in tables and figures, but reflect analysis of all available time points. Kaplan-Meier survival curves were plotted and statistical differences assessed using the Gehan-Breslow test. The hematological data from survivors in the *HS-50_BV_* and *HS-65_BV_+VP series* were pooled; analyzing changes from baseline to 72 hours post-resuscitation within each treatment group by paired *t*-test and differences between groups by unpaired *t*-test. The data were presented as means ± SD unless otherwise stated. A two-tailed probability value of *p*<0.05 was considered significant.

## Results

No unexpected adverse events occurred. Demise occurred attributed to hemorrhagic shock consequent to hemodynamic compromise during the acute phase and to organ dysfunction during the 72 hour observation interval.

### Baseline

No significant differences between EPO and control groups were observed at baseline within each series as shown on Table 1 and on each successive tables and figures, except for RVSWI in *HS-50_BV_*.

**Table 1:** Baseline Characteristics.

	*HS-50_BV_*		*HS-65_BV_*		*HS-65_BV_+VP*	
Variable	CTR	EPO	CTR	EPO	CTR	EPO
**n**	12	12	12	12	12	12
**Weight (kg)**	39.6±3.7	37.5±4.2	34.6±1.5	35.4±1.2	39.4±2.4	38.1±2.5
**Preparation Time (min)**	170±36	174±41	123±59	114±21	119±22	113±21
**Temperature (°C)**	38.1±0.4	38.0±0.4	38.0±0.3	38.0±0.2	38.2±0.3	38.2±0.2
**Respiration Rate (bpm)**	31±5	31±4	35±2	36±1	36±2	35±2
**End-tidal CO_2_ (mmHg)**	38±2	38±2	40±2	42±2	38±3	37±3
**Mean Arterial Pressure (mmHg)**	62±8	62±8	59±6	63±9	61±7	68±9
**Cardiac Index (ml/min·m^-^^2^)**	4.6±0.6	4.4±1.1	3.9±0.6	4.2±1.1	4.8±0.8	4.9±0.8
**Heart Rate (bpm)**	98±20	92±9	98±10	106±16	105±20	100±12
**Blood Withdrawal Index (ml/m^2^)**	1398±70	1370±43	1909±162	1972±316	1814±39	1796±39

Values are mean ± SD. *HS-50_BV_*, blood volume withdrawal 50%; *HS-65_BV_*, blood volume withdrawal 65%; *HS-65_BV_+VP*, blood volume withdrawal 65% and vasopressin infusion. CTR, control; EPO, erythropoietin. There were no statistically significant differences between groups within each series.

### EPO plasma levels

**Figure 3:**
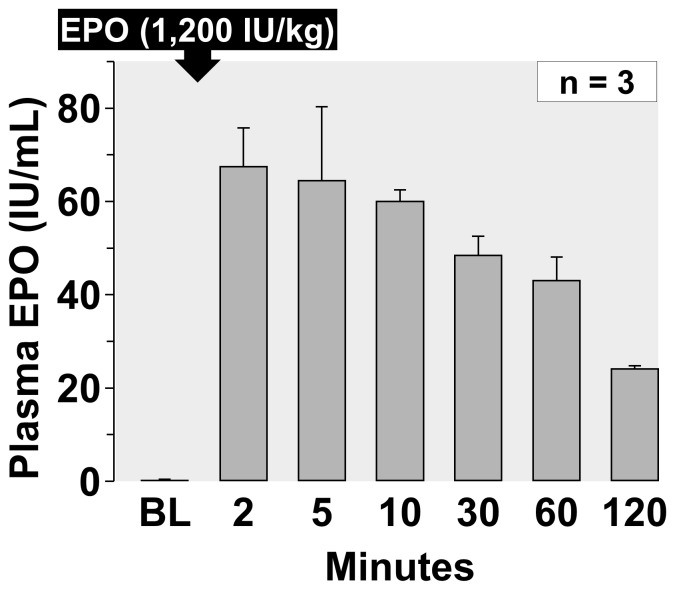
Plasma levels of EPO measured in 3 representative experiments from the *HS-50_BV_* series. Values are mean ± SEM.

Plasma levels of EPO averaging 3 representative experiments from series *HS-50_BV_* are shown in Figure 3 confirming the adequacy of the intraosseous route yielding levels in excess of 20 U/ml for at least 120 minutes after administering 1,200 U/kg.

### Resuscitation and survival

The initial resuscitation rate for all three series and subsequent 72 hour survival for *HS-50_BV_* and *HS-65_BV_ +VP* are shown in Figure 2. For the EPO and the control group combined; *HS-50_BV_* resulted in 88% initial resuscitation and 60% survival; *HS-65_BV_* resulted in only 25% initial resuscitation (noticing that demise started after removing more than 50% of the blood volume); and *HS-65_BV_ +VP* resulted in 92% initial resuscitation (preventing demise after exceeding 50% of blood volume withdrawal) and 83% survival. EPO had no effect on initial resuscitation or subsequent survival in any of the three series.

### Hemodynamic and myocardial function

**Figure 4:**
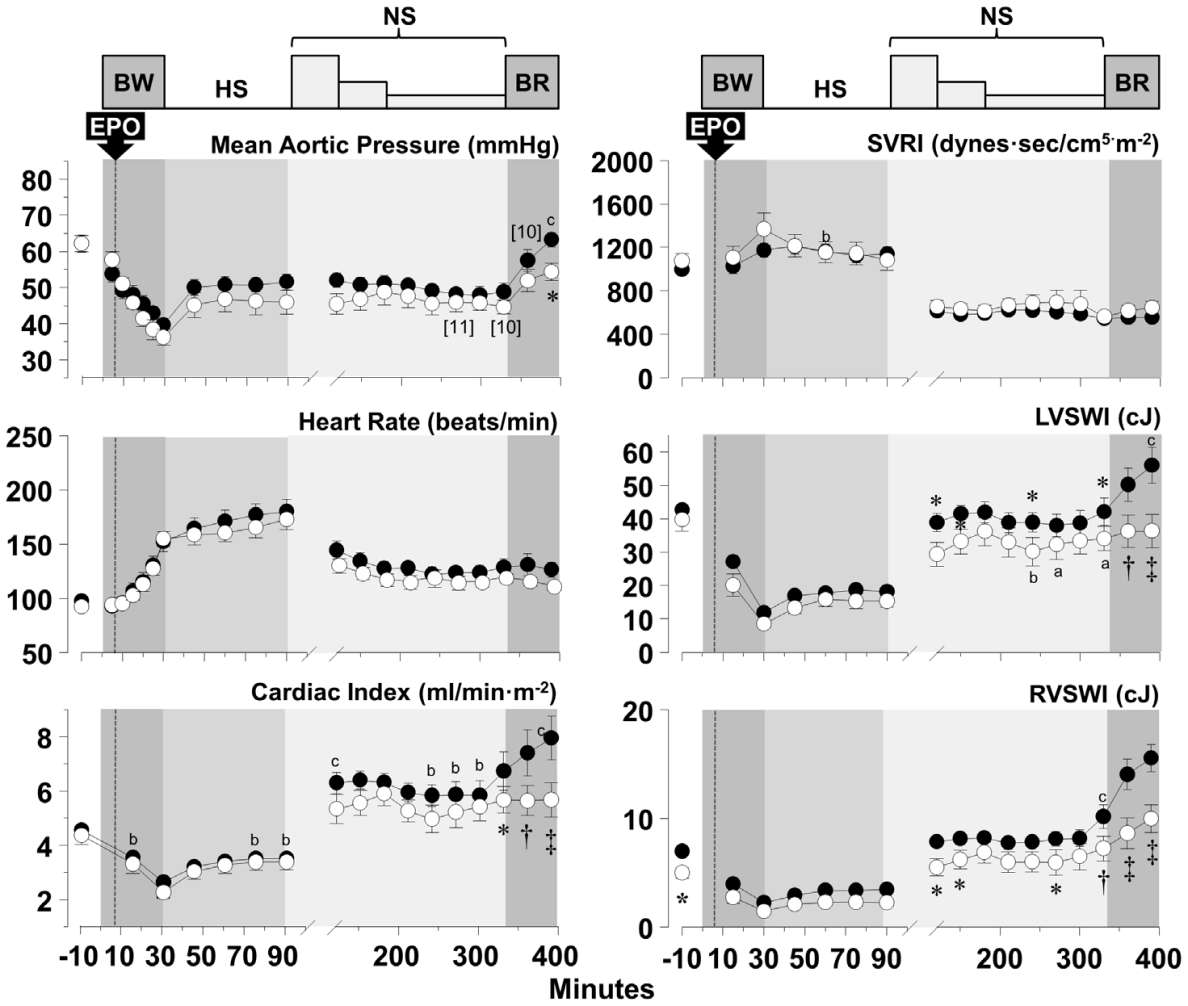
Hemodynamic and myocardial effects of EPO (open circles, n = 12) and vehicle control (closed circles, n = 12) in series *HS-50_BV_*. Numbers in brackets indicate when the number of animals decreased from the preceding time point consequent to death of the animal. BL, baseline; BW, blood withdrawal; HS, hemorrhagic shock; NS, normal saline; BR, blood reinfusion; Ao, aortic pressure; SVRI, systemic vascular resistance index; LVSWI, left ventricular stroke work index; RVWI, right ventricular stroke work index. Values are shown as mean ± SEM. Differences between groups were analyzed by two-way repeated measures ANOVA. There were no overall significant treatment effects. However, there were overall statistically significant interactions between treatment and time for Ao mean (*p* = 0.033), cardiac index (*p*<0.001), LVSWI (*p* = 0.001), and RVSWI (*p*<0.001). *p≤0.05, †*p*≤0.01, and ‡*p*≤0.001 denote statistically significant differences between groups at the specified time points. ^a^ *p*≤0.05, ^b^ *p*≤0.01, and ^c^ *p*≤0.001 denote significant differences *vs* baseline using the Holm-Sidak test for multiple comparisons showing the differences only when they occurred in one of the two groups.

**Figure 5:**
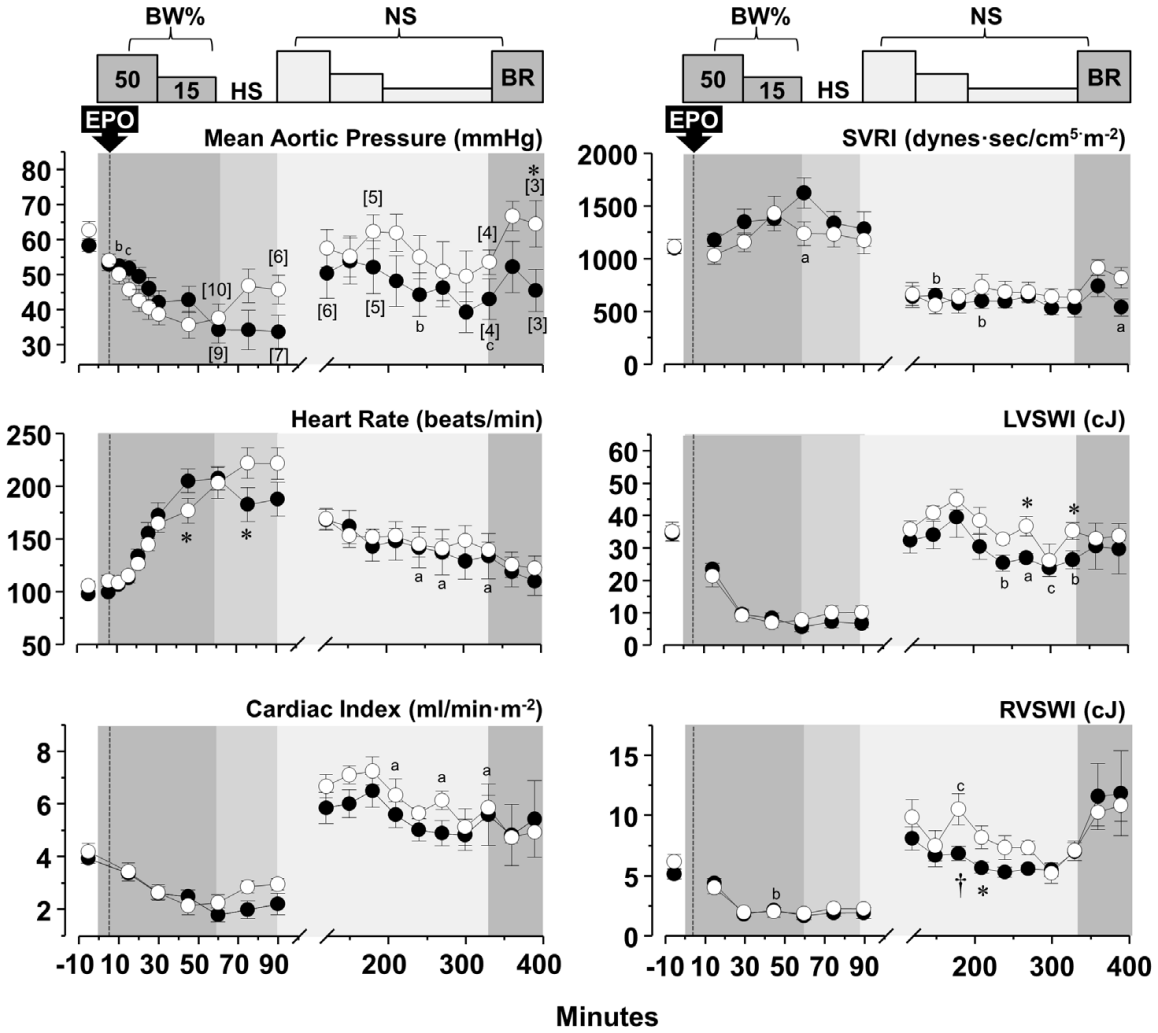
Hemodynamic and myocardial effects of EPO (open circles, n = 12) compared with vehicle control (closed circles, n = 12) in series *HS-65_BV_*. Numbers in brackets indicate when the number of animals decreased from the preceding time point consequent to death of the animal. BL, baseline; BW, blood withdrawal; HS, hemorrhagic shock; NS, normal saline; BR, blood reinfusion; Ao, aortic pressure; SVRI, systemic vascular resistance index; LVSWI, left ventricular stroke work index; RVWI, right ventricular stroke work index. Values are shown as mean ± SEM. Differences between groups were analyzed by two-way repeated measures ANOVA. There were no overall significant treatment effects. However, there was an overall statistically significant interaction between treatment and time for Ao mean (*p* = 0.002). *p≤0.05, †*p*≤0.01, and ‡*p*≤0.001 denote statistically significant differences between groups at the specified time points. ^a^ *p*≤0.05, ^b^ *p*≤0.01, and ^c^ *p*≤0.001 denote significant differences *vs* baseline using the Holm-Sidak test for multiple comparisons showing the differences only when they occurred in one of the two groups.

**Figure 6:**
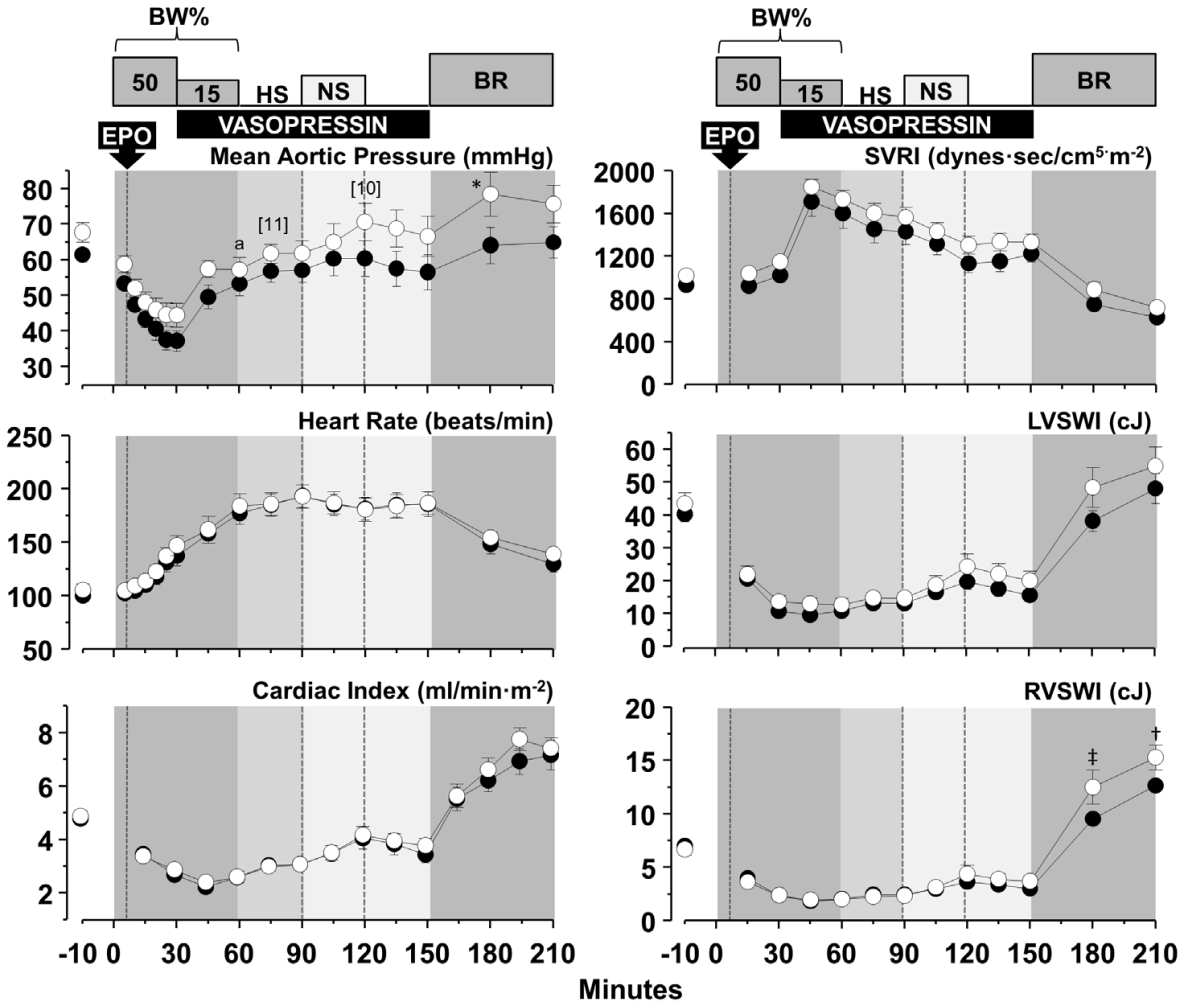
Hemodynamic and myocardial effects of EPO (open circles, n = 12) compared with vehicle control (closed circles, n = 12) in *HS-65_BV_+VP*. Numbers in brackets indicate when the number of animals decreased from the preceding time point consequent to death of the animal. BL, baseline; BW, blood withdrawal; HS, hemorrhagic shock; NS, normal saline; BR, blood reinfusion; Ao, aortic pressure; SVRI, systemic vascular resistance index; LVSWI, left ventricular stroke work index; RVWI, right ventricular stroke work index. Values are shown as mean ± SEM. Differences between groups were analyzed by two-way repeated measures ANOVA. There was an overall statistically significant treatment effect for LVSWI (*p* = 0.035) and an overall statistically significant interaction between treatment and time for Ao mean (*p* = 0.002). *p≤0.05, †*p*≤0.01, and ‡*p*≤0.001 denote statistically significant differences between groups at the specified time points. ^a^ *p*≤0.05, ^b^ *p*≤0.01, and ^c^ *p*≤0.001 denote significant differences *vs* baseline using the Holm-Sidak test for multiple comparisons showing the differences only when they occurred in one of the two groups.

Blood removal triggered a chronotropic response that attenuated reductions in cardiac index and aortic blood pressure yielding a relatively stable hemodynamic state after completion of blood removal despite marked reduction in left and right ventricular work indexes, attributed mainly to reduced preload (Figures 4–6). Administration of normal saline, three-fold the volume of blood removed in series *HS-50_BV_* and *HS-65_BV_*, markedly increased cardiac index (to levels higher than baseline), normalized left and right ventricular work indexes, and reduced the chronotropic response without substantial change in aortic pressure (Figures 4 and 5). Administration of vasopressin in *HS-65_BV_+VP* with or without normal saline (equal to half the volume of blood removed) increased systemic vascular resistance and aortic pressure with a modest increase in cardiac index and left and right stroke work indexes (Figure 6). Blood reinfusion maintained or increased cardiac index, aortic pressure, and work indexes in *HS-50_BV_* and *HS-65_BV_*; whereas in *HS-65_BV_+VP* the predominant effect was increase in cardiac index and work indices (Figure 4–6). Relatively minor effects that varied contingent on the series were observed in relation to EPO. In *HS-50_BV_*, EPO appeared to blunt the hemodynamic and myocardial response to HS and the subsequent fluid resuscitation and blood reinfusion interval (Figure 4). The opposite effect was observed in *HS-65_BV_* and *HS-65_BV_+VP* in which favorable hemodynamic and myocardial response were more prominent in the EPO group during HS and the subsequent fluid resuscitation and blood reinfusion intervals (Figure 5 and 6).

### Oxygen metabolism and lactatemia

**Figure 7:**
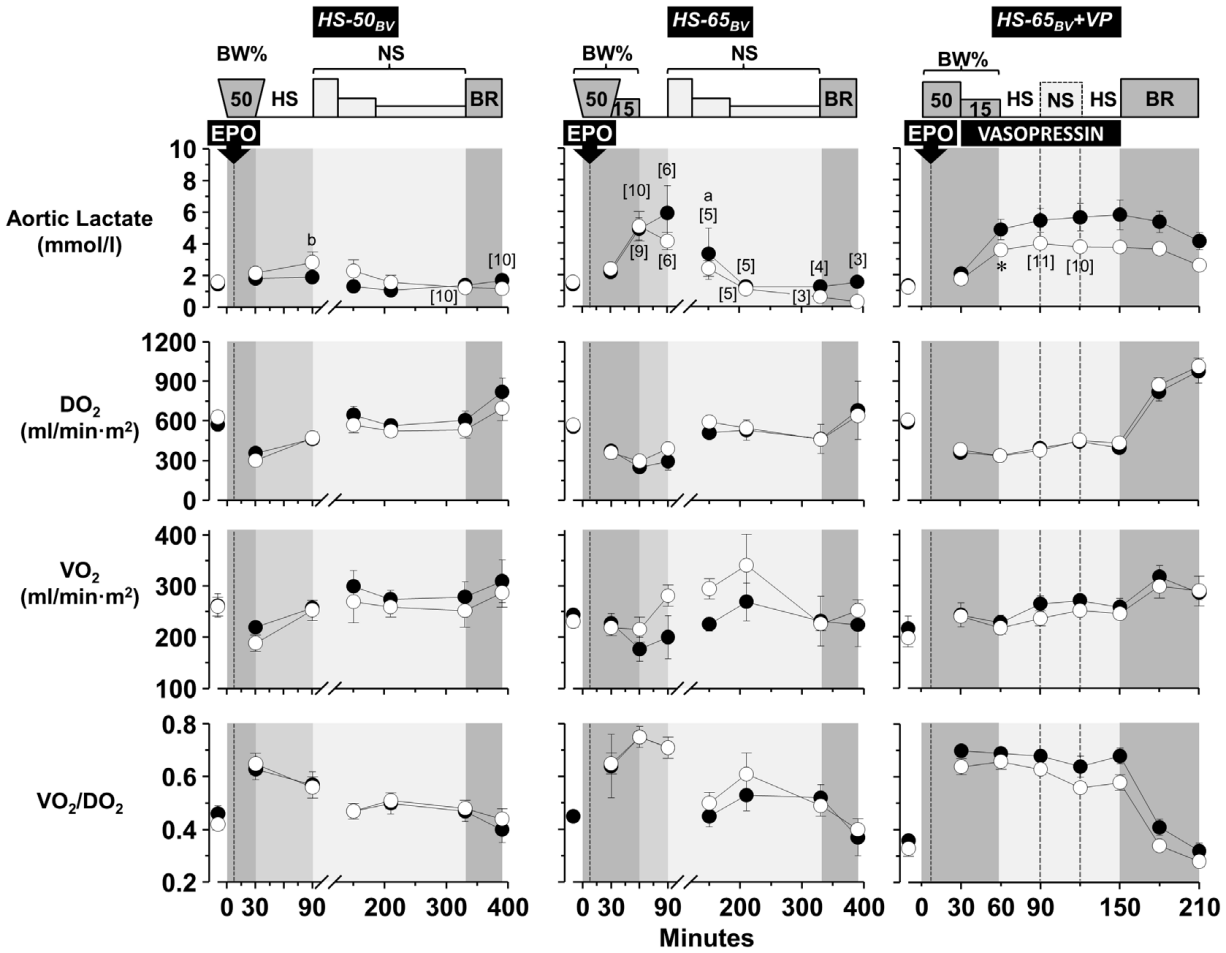
Metabolic effects of EPO (open circles) compared with vehicle control (closed circles) in series *HS-50_BV_*, *HS-65_BV_*, and *HS-65_BV_+VP*. Numbers in brackets indicate when the number of animals decreased from the preceding time point. BW, blood withdrawal; HS, hemorrhagic shock; NS, normal saline; BR, blood reinfusion. Values are shown as mean ± SEM. Differences between groups were analyzed by two-way repeated measures ANOVA for each series separately. There were no overall significant treatment effects. However, there was an overall statistically significant interaction between treatment and time for lactate in series *HS-65_BV_+VP* (*p* = 0.007). *p≤0.05 denotes statistically significant differences between groups at the specified time points. ^a^ *p*≤0.05 and ^b^ *p*≤0.01 denote significant differences *vs* baseline using the Holm-Sidak test for multiple comparisons showing the differences only when they occurred in one of the two groups.

The chronotropic response along with the expected increase in systemic oxygen extraction in *HS-50_BV_* allowed adequate adaptation to HS evidenced by minimal lactatemia and high resuscitation and survival rates (Figure 7 and Figure 2, *HS-50_BV_*). Greater blood volume removal in *HS-65_BV_* exhausted the adaptive response evidenced by higher systemic oxygen extraction, higher levels of lactic acid, and substantial demise after removing more than 50% of the blood volume (Figure 7 and Figure 2, *HS-65_BV_*). Use of vasopressin in *HS-65_BV_+VP* enabled survival despite similar exhaustion of the adaptive response and was attributed to maintaining a higher aortic pressure required for coronary perfusion (Figure 7 and Figure 2, *HS-65_BV_+VP*). Relatively minor metabolic effects related to EPO were observed, highlighting an attenuation of lactate increase in *HS-65_BV_+VP* (Figure 7).

### Myocardial metabolism

In *HS-65_BV_*, potential myocardial metabolic effects produced by severe HS were assessed measuring myocardial oxygen, lactate, and pCO_2_ differences across the coronary circuit. As shown in Table 2, HS was not associated with myocardial ischemia (despite ischemia in other organs evidenced by lactatemia), with EPO and control groups behaving similarly.

**Table 2:** Myocardial Metabolic Effects of EPO in *HS-65_BV_*.

	Baseline	End BW 50%	End BW 15%	End HS	NS	End NS	End BR
	−10 min	30 min	60 min	90 min	210 min	330 min	390 min
**Ao O_2_ Content (ml/dl)**							
CTR	14.1±1.2	13.9±1.9	13.7±1.3[^9^]	12.6±2.7[^6^]	9.3±1.4[^5^]	8.1±1.3[^4^]	12.2±1.8[^3^]
EPO	13.6±1.3	13.6±1.4	13.3±1.4[^10^]	13.3±1.4[^6^]	8.8±1.0[^5^]	7.9±0.9[^3^]	13.0±1.0
**GCV O_2_ Content (ml/dl)**							
CTR	3.6±1.6	3.5±1.5	2.5±0.6^c^	2.7±0.6^c^	3.0±0.4^c^	1.3±1.5	4.01±1.4
EPO	2.8±0.8	2.8±0.8	2.6±1.3	2.6±0.3	2.9±0.9	2.9±0.3	4.04±1.3
**O_2_ Extraction Ratio ([Ao-GCV]/Ao)**							
CTR	0.75±0.10	0.76±0.09	0.81±0.05	0.78±0.04	0.67±0.03	0.66±0.07	0.67±0.08
EPO	0.80±0.05	0.79±0.05	0.80±0.09	0.80±0.02	0.66±0.11	0.63±0.04	0.69±0.11
**GCV-Ao Lactate Gradient (mmol/l)**							
CTR	−0.8±0.5	−1.2±0.7	−0.9±0.7	−1.1±0.6	0.1±0.3	−0.0±0.7	−0.5±0.5
EPO	−0.9±0.5	−1.5±0.5	−1.6±0.4	−1.3±0.8	0.0±0.4	−0.2±0.1	0.0±0.0
**GCV-Ao pCO_2_ Gradient (mmHg)**							
CTR	13±4	15±4	19±4^a^	16±4	12±6	11±9	10±3
EPO	15±3	14±2	16±10	18±3	13±4	9±2	12±3

Numbers in brackets indicate when the sample size decreased from the initial twelve animals. BW, blood withdrawal; HS, hemorrhagic shock; NS, normal saline; BR, blood reinfusion; EPO, erythropoietin; CTR, control; Ao, aorta; GCV, great cardiac vein. Values are mean ± SD. The data was analyzed using two-way repeated measures ANOVA. There were no overall significant treatment effects and no overall statistically significant interactions between treatment and time. ^a^ *p≤*0.05; ^c^ *p≤*0.001 denote significant differences *vs* baseline using the Holm-Sidak test for multiple comparisons showing the differences only when they occurred in one of the two groups.

### Effects of fluid resuscitation

**Figure 8:**
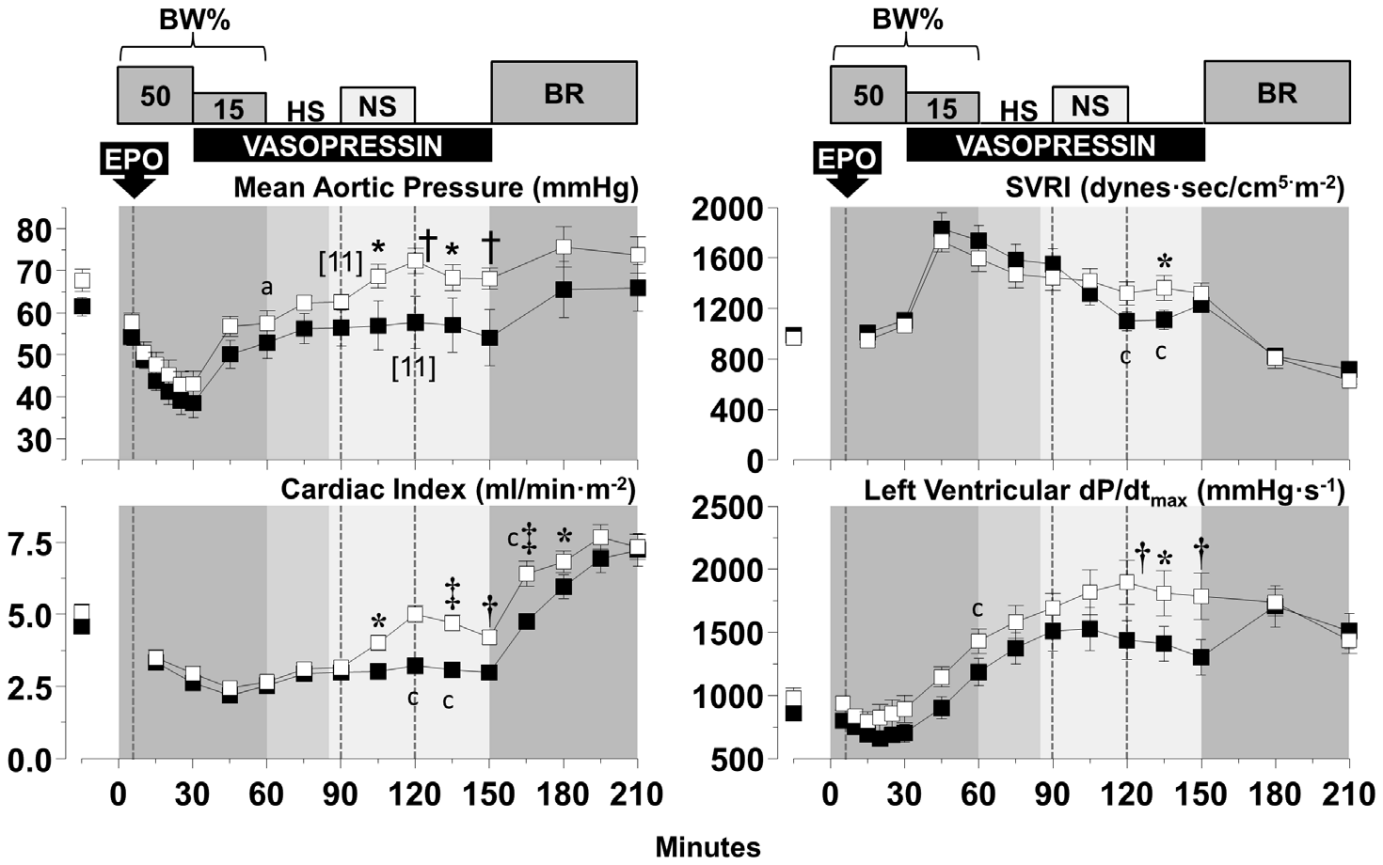
Hemodynamic effects of fluid resuscitation (open symbols, n = 12) and no fluid resuscitation (closed symbols, n = 12) in series *HS-65_BV_+VP*. Numbers in brackets indicate when the number of animals decreased from the preceding time point consequent to death of the animal. BL, baseline; BW, blood withdrawal; HS, hemorrhagic shock; NS, normal saline; BR, blood reinfusion; Ao, aortic pressure; SVRI, systemic vascular resistance index. Values are shown as mean ± SEM. Differences between groups were analyzed by two-way repeated measures ANOVA. There was an overall statistically significant treatment effect for cardiac index (*p* = 0.021). There were also overall statistically significant interactions between treatment and time for cardiac index (*p*<0.001) and SVRI (*p*<0.001). *p≤0.05, †*p*≤0.01, and ‡*p*≤0.001 denote statistically significant differences between groups at the specified time points. ^a^ *p*≤0.05, and ^c^ *p*≤0.001 denote significant differences *vs* baseline using the Holm-Sidak test for multiple comparisons showing the differences only when they occurred in one of the two groups.

The effect of low-volume fluid administration was assessed in the *HS-65_BV_+VP series* and shown in Figure 8 and Table 3. Fluid administration promoted an increase in cardiac index, mean aortic pressure, and left ventricular dP/dt_max_ (Figure 8) accompanied by attenuation of systemic oxygen extraction and faster normalization of lactic acidosis (Table 3). Of the 12 animals that received fluids, 11 were resuscitated and remained alive at 72 hours. Of the 12 animals that did not receive fluids, 11 were also resuscitated but only 9 were alive at 72 hours; a difference however that was not statically significant.

**Table 3:** Metabolic Effect of Fluid Resuscitation in *HS-65_BV_+VP*.

	Baseline	End BW 65%	HS/NS	End HS	End BR
	−10 min	60 min	120 min	150 min	210 min
**Aortic Lactate (mmol/l)**					
NS	1.3±0.7	3.9±1.0	4.0±1.0[^11^] *	3.9±0.9*	2.7±1.0*
No NS	1.3±0.5	4.0±2.4	5.6±3.1[^11^]	5.8±3.3	4.1±1.9
**Aortic pH**					
NS	7.48±0.05	7.41±0.06	7.33±0.03	7.37±0.04	7.40±0.03
No NS	7.47±0.04	7.38±0.06	7.33±0.06	7.34±0.02	7.37±0.02
**Aortic O_2_ Content (ml/dl)**					
NS	12.7±1.3	13.3±1.3	10.1±1.1^‡^	10.9±1.0*^c^	13.0±0.6
No NS	12.1±2.1	12.8±1.8^a^	12.4±1.7	12.4±1.5	14.1±1.9^c^
**Venous O_2_ Content (ml/dl)**					
NS	8.1±1.2	4.6±1.1	4.7±0.7	4.7±0.9	9.2±0.7
No NS	8.0±1.4	4.0±1.3	4.2±1.6	3.8±1.6	9.8±2.3
**VO_2_/DO_2_ (ratio)**					
NS	0.36±0.10	0.66±0.08	0.54±0.04*	0.57±0.07*	0.29±0.04*^a^
No NS	0.33±0.09	0.69±0.08	0.67±0.11	0.70±0.11	0.31±0.12

Numbers in brackets indicate when the sample size decreased from the initial twelve animals. Values are mean ± SD. BW, blood withdrawal; HS, hemorrhagic shock; NS, normal saline; BR, blood reinfusion; VO_2_/DO_2_, oxygen consumption divided by oxygen delivery. The data was analyzed using two-way repeated measures ANOVA. There were overall statistically significant interactions between treatment and time for aortic pH (*p* = 0.043), aortic O_2_ content (*p≤*0.001), and VO_2_/DO_2_ ratio (*p≤*0.001). There was no overall statistically significant treatment effect. *p≤0.05 and **^‡^** *p≤*0.001 denote statistically significant differences between groups at the specified time point. ^a^ *p≤*0.05 and ^c^ *p≤*0.001 denote statistically significant differences *vs* baseline using the Holm-Sidak test for multiple comparisons showing the differences only when they occurred in one of the two groups.

### Organ injury and function

The post-resuscitation effect on various organs was assessed daily in *HS-65_BV_+VP*. As shown in Table 4, EPO treated animals had an attenuated increase in AST, ALT, troponin I, and less neurological deficit at some point during the post-resuscitation phase. There were statistically insignificant differences suggesting less kidney injury in the EPO group. There was no difference in the percentage of lung water at 72 hours between EPO and control pigs (81.0±0.6% *vs* 81.8±1.0%).

**Table 4:** Effect of EPO on Organ Function in *HS-65_BV_+VP*.

	Baseline	Post-Resuscitation		
	−10 min	24 h	48 h	72 h
**Creatinine (mg/dl)**				
CTR	1.3±0.2	1.5±1.4	1.4±1.7 [^11^]	0.9±0.1^b ^ [^10^]
EPO	1.0±0.2	0.9±0.2 [^10^]	0.9±0.2	0.9±0.1
**Blood Urea Nitrogen (mg/dl)**				
CTR	10±5	22±22	15±24	7±2
EPO	9±3	9±3	7±3	7±2
**AST (U/l)**				
CTR	35±7	400±501^c^	188±204	113±118
EPO	32±6	168±101^†^	85±43	67±26
**ALT (U/l)**				
CTR	51±10	126±109^c^	107±51^c^	100±36
EPO	53±8	82±24*	86±26	58±23^c^
**Troponin I (ng/ml)**				
CTR	0.22±0.18	1.47±2.40^c^	0.48±1.06	0.22±0.24
EPO	0.15±0.09	0.41±0.29	0.20±0.47	0.12±0.09*
**Neurologic Deficit Score**				
CTR	0±0	26±45^a^	15±45	2±6
EPO	0±0	13±35	0±0	0±0

Numbers in brackets indicate when the sample size decreased from the initial twelve animals. Values are mean ± SD. CTR, control; EPO, erythropoietin. The data was analyzed using two-way repeated measures ANOVA. There was no overall significant treatment effect and no overall statistically significant interactions between treatment and time. *p≤0.05 and ^†^ *p≤*0.01 denote statistically significant differences between groups at the specified time points. ^a^ *p≤*0.05, ^b^ *p≤*0.01, and ^c^ *p≤*0.001 denote statistically significant differences *vs* baseline using the Holm-Sidak test for multiple comparisons showing the differences only when they occurred in one of the two groups.

### Plasma cytokines

Plasma IL-6, IL-8, IL-10, and TNF-α were measured in the *HS-65_BV_+VP* series, analyzing the effects of EPO (Table 5) and the effects of fluid resuscitation (Table 6). Overall there was a time effect with increases in IL-8 and IL-10 by the end of blood reinfusion reversing to baseline by 72 hours. Most prominently, EPO was associated with an increase in IL-10 by the end of blood reinfusion (Table 5). Administration of normal saline blunted increases in IL-8 and in IL-10 (Table 6).

**Table 5:** Effect of EPO on Plasma Cytokines in *HS-65_BV_+VP*.

	Baseline	End BR	Post-Resuscitation	
	−10 min	210 min	24 h	72 h
**IL-6 (pg/ml)**				
CTR	42±28 [34]; [25]	79±55 [63; 34]	794±2515 [59, 53]	115±152 [66; 43]
EPO	30±28 [16]; [43]	106±73 [94; 57]	58±72 [25]; [30]	34±24 [26]; [28]
**IL-8 (pg/ml)**				
CTR	18±13 [14]; [22]	34±4 [24]; [27]	17±22 [6]; [15]	10±5 [12]; [8]
EPO	18±14 [15;15]	18±12 [15]; [17]	7±4 [5]; [3]	8±5 [7]; [2]
**IL-10 (pg/ml)**				
CTR	23±50; [8;8]	70±81 [33]; [28]	16±14 [11]; [9]	11±9 [8]; [4]
EPO	12±7 [12]; [9]	207±281 [70; 174]^b†^	15±16 [7]; [8]	10±6 [8]; [3]
**TNF-α (pg/ml)**				
CTR	31±12 [30]; [9]	31±9 [33]; [10]	24±13 [24]; [11]	29±11 [28]; [12]
EPO	24±7 [21]; [6]	37±26 [27]; [15] ^a^	20±8 [18]; [4]	24±5 [22]; [5]

Samples were available for each of the 12 control (CTR) pigs including the 10 that survived at 72 hours; but, for only 10 of the erythropoietin (EPO) treated pigs, which included all the survivors. Values are mean ± SD showing also the median with interquartile range in brackets as values for several time events were not normally distributed. BR, blood reinfusion; IL, interleukin; TNF-α, tumor necrosis factor-α. The data was analyzed using two-way repeated measures ANOVA. There was no overall statistically significant treatment effect. There was a statistically significant time effect for IL-8 (*p = *0.011), IL-10 (*p≤*0.001) and TNF-α (*p = *0.006) and there was a borderline statistically significant interactions between treatment and time for IL-10 (*p = *0.062). ^†^ *p≤*0.003 denotes a statistically significant difference between groups at the specified time point. ^a^ *p≤*0.05, ^b^ *p≤*0.001 denote statistically significant differences from baseline within each group using the Holm-Sidak test for multiple comparisons.

**Table 6:** Effect of Fluid Resuscitation on Plasma Cytokines in *HS-65_BV_+VP*.

	Baseline	End BR	Post-Resuscitation	
	−10 min	210 min	24 h	72 h
**IL-6 (pg/ml)**				
NS	30±22 [23;23]	62±27 [57; 24]	40±23 [38]; [20]	42±25 [38]; [33]
No NS	43±33 [33]; [45]	120±77 [101; 68]	880±2620 [83; 132]	115±164 [69; 61]
**IL-8 (pg/ml)**				
NS	16±14 [10]; [17]	16±11 [17;17]	9±9 [5]; [4]	8±4 [6]; [5]
No NS	20±11 [19]; [13]	37±42 [32]; [27] *	16±23 [5]; [9]	11±5 [11]; [9]
**IL-10 (pg/ml)**				
NS	25±53 [9]; [11]	84±120 [38]; [35]	8±4 [7]; [4]	10±6 [9]; [3]
No NS	11±5 [9]; [6]	181±262 [67; 206]^a^*	23±17 [14]; [28]	12±9 [8]; [3]
**TNF-α (pg/ml)**				
NS	28±15 [21]; [16]	30±10 [29]; [10]	26±13 [25]; [17]	30±11 [27]; [13]
No NS	27±5 [27]; [5]	38±24 [31]; [16]	18±7 [19]; [5]	23±4 [22]; [3]

Samples were available for all 11 pigs in each group that survived the initial 24 hours and for all the 9 that survived at 72 hours from the group without fluid resuscitation. Values are mean ± SD showing also the median with interquartile range in brackets as values for several time events were not normally distributed. BR, blood reinfusion; NS, normal saline; IL, interleukin; TNF-α, tumor necrosis factor-α. The data was analyzed using two-way repeated measures ANOVA. There was no overall statistically significant treatment effect. There was a statistically significant time effect for IL-8 (*p* = 0.008), IL-10 (*p≤*0.001), and TNF-α (*p* = 0.008). *p≤0.05 denotes a statistically significant difference between groups at the specified time points. ^a^ *p≤*0.001 denotes a statistically significant difference from baseline using the Holm-Sidak test for multiple comparisons.

### Hematological effects

Pooled data from animals that survived in the *HS-50_BV_* and *HS-65_BV_+VP* series (18 animals in control group and 17 in EPO group) showed no differences between treatment groups at baseline with cell counts within normal for swine [29]. Red blood cell count and hematocrit increased relative to baseline in both groups at 72 hours post-resuscitation but to a greater extent in the EPO group (Table 7). Over the same time interval, platelet count increased but only in the EPO group whereas the white blood cell count remained unchanged.

**Table 7:** Hematological Effects of EPO in *HS-50_BV_* and *HS-65_BV_+VP*.

		Baseline	72 h Post-Resuscitation
**Red Blood Cells (10^6^/µl)**	CTR	5.5±0.4[^18^]	6.0±0.8^a^
	EPO	5.6±0.6[^17^]	6.8±0.7*^c^
**Hematocrit (%)**	CTR	31.5±1.7	34.4±4.8^a^
	EPO	31.6±2.1	39.3±4.5*^c^
**White Blood Cells (10^3^/µl)**	CTR	18.0±4.0	20.9±4.9
	EPO	20.0±3.5	21.0±5.5
**Platelets (10^3^/µl)**	CTR	312±68	332±106
	EPO	304±85	397±164^a^

Values are mean ± SD. CTR, control; EPO, erythropoietin. Data was pooled from *HS-50_BV_* and *HS-65_BV_+VP series*. Numbers in bracket indicate pooled sample size. Unpaired *t*-test was used to compare differences in the pooled hematological data between treatment groups at given time points. *p≤0.05. Paired *t*-test was used to compare pooled hematological data from *HS-50_BV_* and *HS-65_BV_+VP series* at baseline and post-resuscitation within each treatment group. ^a^ *p≤*0.05 and ^c^ *p≤*0.001. There were no statistically significant differences between groups at baseline.

## Discussion

The present study failed to demonstrate a beneficial (or detrimental) effect of EPO on initial resuscitability or subsequent survival in a swine model of HS regardless of its severity. The work was conducted in three consecutive HS series modeling mild severity, high severity with high fatality despite aggressive fluid resuscitation, and high severity with low fatality associated with vasopressin infusion and low-fluid or no-fluid resuscitation. EPO in the last series featuring high severity with low fatality increased plasma levels of the anti-inflammatory cytokine IL-10, attenuated lactatemia, and lessened transient injury to the liver, heart, and brain based on enzyme release and clinical neurological deficit. In addition, the study addressed several current aspects of HS management showing the efficacy of vasopressin infusion and restrictive fluid resuscitation.

### EPO Effect

The dose of EPO chosen for the present experiments (1,200 U/kg) was extrapolated from a dose previously used in a study targeting sudden cardiac arrest victims (90,000 U) [21]. A lower dose (12,000 U) was deemed effective in a pilot human assessing potential protection after acute myocardial infarction [30]. In the present study, we confirmed that the chosen EPO dose – delivered through the intraosseous route – reached the bloodstream (Figure 3) attaining plasma levels that exceeded 30 U/ml for the initial 60 minutes decreasing to approximately 20 U/ml after 120 minutes. Only a brief exposure to EPO is required to trigger a sustained protective effect during HS [31] and levels above 5 U/ml are sufficient to elicit cytoprotection [32]. Accordingly, the lack of effects of EPO on resuscitability and survival occurred despite a sustained presence of EPO in the bloodstream and evidence of biological activity given the significant increase in hematocrit and platelets after 72 hours (Table 5).

The lack of impact on initial resuscitability from HS likely reflected the mechanism of death. Animals died preceded by progressive reductions in blood pressure that at some point precipitously compromised coronary perfusion and cardiac function. However, there was no evidence of protracted myocardial ischemia before demise upon which EPO could have exerted an acute “protective” effect, as previously reported during resuscitation from cardiac arrest [20]. The lack of myocardial ischemia was shown in series *HS-65_BV_* (the series with the highest HS severity and mortality), in which the lactate gradient across the coronary circuit was negative (i.e., lactate utilization) and the PCO_2_ gradient was not increased [25], both indicating absence of myocardial ischemia (Table 2). At the same time, substantial systemic lactic acidosis developed in series *HS-65_BV_* and series *HS-65_BV_+VP*, indicating critical reductions in oxygen delivery prompting anaerobic metabolism in other tissues. In *HS-65_BV_+VP*, EPO attenuated increases in lactic acid, consistent with a beneficial effect at the mitochondrial level as we have reported in a rat model of cardiac arrest [20]. Likewise, there was attenuation of markers of organ injury in EPO treated pigs in the *HS-65_BV_+VP* series. The protective effects at the cell level reported by us [20] and others [32] in rats involve at the very least Akt activation (phosphorylation); a kinase that plays a key role in cell survival and other adaptive responses including mitochondrial bioenergetic function and enhanced myocardial contractility [33], [34]. In addition, plasma IL-10 was higher in pigs that received EPO at the end of blood reinfusion. IL-10 mediates anti-inflammatory effects and EPO can increase production of IL-10 [35]–[37]; pointing to the pleiotropic effect of EPO and mediation of additional effects that could be potentially beneficial for HS. The levels of IL-6 and IL-8 (pro-inflammatory cytokines) were lower at 24 and 72 hours in pigs treated with EPO but the differences were not statistically significant.

Studying the potential clinical relevance of these effects at the organ level was beyond the scope of the present study. In clinical settings, patients are exposed to repetitive injuries after stabilization (i.e., biological, infectious, sterile, etc.) and information on whether organ susceptibility to subsequent injuries could be ameliorated by EPO would be of substantial clinical interest.

### Hemodynamic Response to Hemorrhagic Shock

A prominent (adaptive) chronotropic response maintained the cardiac index in *HS-50_BV_* at ∼80% of baseline, enabling increases in oxygen extraction to preserve aerobic metabolism and thereby yielding only minor increases in lactic acid. In series *HS-65_BV_* and *HS-65_BV_+VP*, however, the cardiac index was maintained at ∼62% of baseline and the oxygen extraction reached its maximum level prompting systemic lactic acidosis; most likely from tissues excluded from the circulation following redistribution of blood flow towards vital organs. Accordingly, in this swine model, the critical transition from “compensated” to “uncompensated” HS occurred after removing 50% of the blood volume.

### Vasopressin

Hemodynamic crises, including HS, trigger the release of vasopressin as part of a prominent neuroendocrine stress response. However, the endogenous vasopressin response is time-limited and exogenous administration has been suggested for hemodynamic stabilization in sepsis [38], [39] and HS [40], [41]. In models of HS, vasopressin was comparably superior to fluid administration or epinephrine [42], [43] and has been proposed as the “preferred” vasopressor agent for hemodynamic stabilization during HS [41]. In a small clinical study in civilians suffering traumatic injury with hypotension, vasopressin infusion reduced the need of resuscitation fluids [44]. Clinicaltrials.gov lists two studies examining the effects of vasopressin in civilians with HS; the AVERT Shock study by Sims, C and the VITRIS study by Wenzel, V. [45].

These reasons prompted us to select vasopressin in our last series (*HS-65_BV_+VP*), with the dose chosen according to a previous study by Voelckel *et al.*, also in swine [42]. The timing for initiation of vasopressin infusion was based on series *HS-65_BV_*, noticing that demise began to occur after removing greater than 50% of the blood volume (Figure 2). The effect of vasopressin infusion on resuscitability was impressive, increasing from 25% (in *HS-65_BV_*) to 92% (in *HS-65_BV_+VP*) for the same degree of blood volume removal (Figure 2). Moreover, 83% of the animals in *HS-65_BV_+VP* were alive at 72 hours and with essentially no organ dysfunction (Table 4).

### Fluid Resuscitation

There is growing consensus that aggressive fluid resuscitation during HS can be detrimental [46]. Coagulation is compromised consequent to dilution of clotting factors and reduced activity by hypothermia and acidosis. Concomitantly, the endogenous fibrinolytic system is activated, tilting the hemostatic balance toward bleeding [47]. Excessive fluid also drives edema formation, which may affect the lungs and other tissues [48]. Large amounts of normal saline can precipitate hyperchloremic acidosis which has been associated with increased risk of renal injury [49] and activation of inflammatory cascades [50]. For this and other (logistic) reasons, resuscitation with minimal or no fluid is gaining acceptance. The Tactical Combat Casualty Care Guidelines recommend fluid administration during HS only when there is altered mental status (in the absence of head injury) and weak or absent peripheral pulses.

In our initial two series (i.e., *HS-50_BV_* and *HS-65_BV_*), we administered normal saline 3 times the amount of blood removed according to the old paradigm resulting in a substantial increase in cardiac index (*via* preload increase) exceeding baseline levels. However, despite high initial resuscitability, the 72 hour survival was less than in the more severe *HS-65_BV_+VP* series suggesting that excess fluid could have been detrimental. In the *HS-65_BV_+VP* series, we examined the current paradigm and administered normal saline half the amount of blood removed in half of the animals and no fluids in the other half observing no differences in resuscitability or survival. Normal saline, however, had as positive effect on cardiac index, aortic pressure, and left ventricular dP/dt_max_ while reducing systemic oxygen extraction and the generation of lactic acid (Figure 8, Table 3). Thus, there was hemodynamic and metabolic benefit elicited by fluid administration which could be critical under conditions of more severe HS and when vasopressin alone is not sufficient.

### Limitations

Extrapolation of the current findings to real-life battlefield resuscitation from hemorrhagic shock is limited by several factors. Upmost is modeling HS without additional tissue injury. Although HS can occur in military and (more so) in civilian settings with low-grade or unassociated tissue injury, HS in the battlefield occurs typically accompanied by substantial tissue injury. Tissue injury compounds the severity of HS partly by the specific organ dysfunction, wound contamination, and amplification of the inflammatory response. In addition, the rate of blood withdrawal was controlled by a predetermined algorithm and did not follow the natural profile of bleeding after injury. The present studies, however, allowed assessing the effects of the studied interventions on severe HS without the confounding effects of tissue injury and uncontrolled bleeding. In subsequent series we incorporated the elements of tissue injury and uncontrolled bleeding by using a liver laceration model. Other limitations include the use of anesthesia, masking some of the physiologic responses to hypovolemic shock. The naturally occurring neuropeptide in swine is lysine vasopressin whereas in humans it is arginine vasopressin (the one used in the present studies). Thus, potency may vary contingent on specific receptors and vascular beds and translation of these findings to humans will need to consider human dose-responses. Despite these limitations, our model reproduced key characteristics of resuscitation from hemorrhagic shock and provided mechanistic information under highly controlled conditions that would be difficult to obtain in a more realistic model.

### Conclusions

EPO given during HS in a swine model of HS failed to alter resuscitability and 72 hour survival regardless of HS severity and concomitant treatment with fluids and vasopressin. EPO, however, attenuated lactate increases and acute injury to the liver, heart, and brain based on enzyme release and neurological deficit scores. The studies also showed that vasopressin infusion with restrictive fluid administration is highly effective for hemodynamic stabilization and subsequent survival.

## Supporting Information

Datafile S1 **Hemodynamic and metabolic data from the three hemorrhagic shock series.**
(XLSX)

Checklist S1 **ARRIVE guidelines checklist.**
(PDF)
